# Capacity debt in palliative care: A case report illustrating longitudinal exhaustion following early engagement

**DOI:** 10.1017/S1478951526102272

**Published:** 2026-04-15

**Authors:** Richa Randhawa

**Affiliations:** Independent Researcher, Palliative Medicine, Jaipur, Rajasthan, India

**Keywords:** Palliative care, patient capacity, serious illness communication, ethical pacing, treatment burden

## Abstract

**Objectives:**

Early engagement in palliative and supportive care is widely promoted as a marker of insight, acceptance, and readiness for shared decision-making. Clinicians, however, frequently observe a paradoxical longitudinal pattern in which patients who initially demonstrate high emotional, cognitive, and decisional engagement later become withdrawn or fatigued despite preserved insight. This case report illustrates such a pattern and interprets it using the concept of capacity debt.

**Methods:**

A longitudinal case description is presented, integrating clinical observation with interpretive analysis informed by literature on patient capacity, emotional labor, cumulative complexity, and serious illness communication.

**Results:**

The patient demonstrated high early engagement in goals-of-care discussions, advance care planning, and emotionally demanding conversations. Over time, she developed marked conversational fatigue and withdrawal without evidence of depression, demoralization, denial, or cognitive impairment. Disengagement appeared temporally related to cumulative engagement demands rather than disease progression alone.

**Significance of results:**

This case illustrates how early intensive engagement may contribute to later disengagement through cumulative depletion of patient capacity. Interpreting this pattern as capacity debt provides a non-pathologizing and ethically grounded explanation, highlighting pacing as a core clinical skill in palliative care.

## Introduction

Early engagement is a cornerstone of contemporary palliative and supportive care. Proactive conversations regarding goals of care, advance care planning, symptom experiences, psychosocial concerns, and existential meaning making are widely advocated soon after diagnosis of serious illness (Morrison and Meier 2004; Sudore et al. 2017; World Health Organization 2020) and are associated with improved alignment of care with patient values and reduced non-beneficial interventions near the end of life (Wright et al. 2008; Mack et al. 2012; Bernacki and Block, 2014).

As a result, early engagement has acquired ethical and institutional significance, often functioning as a marker of insight, preparedness, and collaboration (Elwyn et al. 2012). However, clinicians across palliative care settings frequently observe a paradoxical pattern: patients who initially engage deeply in emotionally demanding conversations later become withdrawn or fatigued despite preserved cognition and mood (Back et al. 2008; Epstein and Street 2011; Kissane 2014).

In the absence of a longitudinal explanatory framework, such disengagement is commonly attributed to depression, demoralization, denial, or disease progression (Block 2001; Clarke and Kissane 2002). These explanations fail to account for withdrawal temporally linked to earlier care intensity and may prompt unnecessary escalation of psychosocial interventions (Rushton 2017). This case report illustrates capacity debt as a clinically and ethically useful interpretive framework.

## Case description

A woman in her early 60s with metastatic cancer was referred to palliative care shortly after diagnosis. She was cognitively intact, emotionally articulate, and highly motivated to engage, actively participating in detailed goals-of-care discussions, requesting prognostic clarification, and initiating advance care planning early in the illness trajectory.

Over subsequent months, she engaged in multiple family meetings and explored existential concerns related to meaning, legacy, and future decision-making. She expressed a desire to “address everything early” and was described as insightful and collaborative. Engagement was frequent, prolonged, and emotionally dense.

As the illness progressed, the symptom burden increased modestly but remained well controlled. There was no evidence of delirium, major depression, demoralization, or cognitive impairment (Block 2001; Clarke and Kissane 2002). Despite this, she declined further in-depth conversations, requested shorter visits, avoided revisiting topics, and expressed fatigue specifically related to discussion rather than physical activity, stating, “I’ve already talked about this too much.”

Family members interpreted her withdrawal as emotional disengagement or loss of hope. However, she continued to demonstrate preserved understanding of her illness and participated coherently in concrete decisions when required. Withdrawal appeared selective to emotionally and cognitively demanding engagement rather than to care itself.

## Clinical interpretation: capacity debt

This pattern of early engagement followed by later withdrawal was not adequately explained by psychopathology, denial, or disease progression, but reflected a temporal relationship between early engagement intensity and later exhaustion.

**Figure 1.**
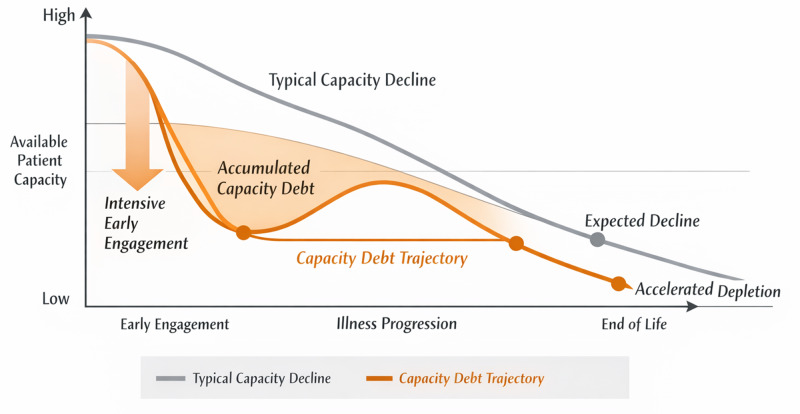
Conceptual trajectory of capacity debt across the illness course. The horizontal axis represents illness trajectory over time, and the vertical axis represents available patient capacity. The dashed line illustrates expected capacity decline due to illness progression alone, while the solid line depicts accelerated depletion resulting from early intensive engagement and accumulation of capacity debt.

Capacity debt refers to the cumulative burden incurred when patients disproportionately expend finite cognitive, emotional, relational, and decisional capacities early in illness, effectively borrowing from future capacity. As illness advances and baseline reserves decline, this debt becomes visible as reduced tolerance for discussion, withdrawal from planning, or preference for silence (Figure 1).

Early engagement required sustained emotional and decisional labor, including confrontation with mortality, regulation of fear, negotiation of family dynamics, and performance of insight. While adaptive initially, this labor accrued internal cost independent of mood or cognition. Disengagement did not reflect denial or loss of insight (Chochinov 2005), but conservation of remaining capacity.

## Discussion

This case illustrates a longitudinal pattern frequently encountered in palliative care but rarely theorized: early, high-intensity engagement followed by later withdrawal despite preserved insight, mood stability, and decisional coherence (Back et al. 2008; Epstein and Street 2011). Existing explanatory frameworks, such as depression, demoralization, denial, or disease progression, do not adequately account for this trajectory (Block 2001; Clarke and Kissane 2002). In contrast, capacity debt offers a clinically coherent and ethically grounded interpretation.

## Capacity debt as a longitudinal construct

Capacity debt describes a temporal phenomenon in which patients expend finite cognitive, emotional, relational, and decisional resources early in serious illness, effectively borrowing strength from their future selves. Unlike static models of capacity or cross-sectional assessments of engagement, capacity debt emphasizes timing, accumulation, and delayed consequence. Engagement that appears adaptive early in illness may carry deferred costs that only become visible as baseline reserves decline. Crucially, capacity debt does not imply error, avoidance, or pathology; it reflects a rational allocation of capacity under conditions of uncertainty. By conceptualizing engagement as a consumptive, time-dependent resource rather than an inexhaustible good, capacity debt provides a longitudinal framework for understanding later disengagement despite preserved insight and decisional coherence. Importantly, capacity debt reframes disengagement not as failure or regression, but as an adaptive response to cumulative relational and existential labor, preserving coherence, dignity, and agency when remaining capacity must be carefully conserved.

**Figure 2.**
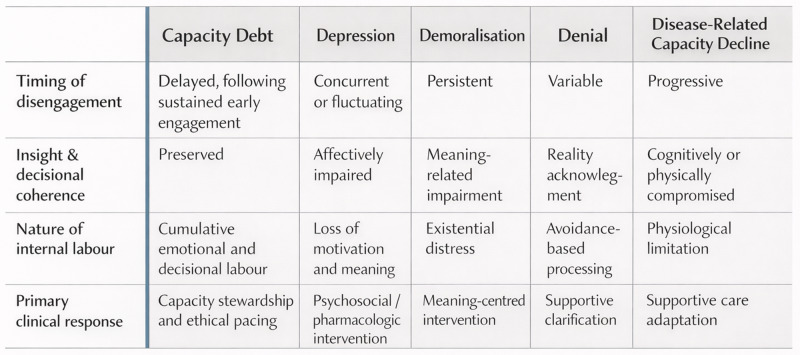
Conceptual boundaries distinguishing capacity debt from alternative explanations for late disengagement. Capacity debt is characterized by delayed disengagement following sustained early engagement, preserved insight and decisional coherence, and cumulative internal emotional and decisional labor. Unlike affective, avoidant, or disease-driven explanations, capacity debt carries distinct ethical implications, reframing disengagement as a signal for pacing and capacity stewardship rather than pathology.

The conceptual boundaries between capacity debt and commonly invoked explanations for late disengagement are summarized in Figure 2. Unlike depression, demoralization, or denial, capacity debt is characterized by preserved insight, delayed emergence following earlier high-intensity engagement, and cumulative internal cognitive and emotional labor, with distinct ethical implications for pacing and capacity stewardship.

Contemporary palliative care strongly emphasizes early engagement, shared decision-making, and advance care planning (Elwyn et al. 2012; Sudore et al. 2017), often operationalized as quality indicators. However, such models frequently assume engagement to be inherently beneficial and indefinitely sustainable. This case demonstrates that engagement itself may constitute labor drawing upon finite and declining patient capacity.

While treatment burden frameworks acknowledge the workload imposed by health-care systems, they focus predominantly on practical and logistical demands (Mair et al. 2012; Shippee et al. 2012; May et al. 2014). Emotional and decisional labor performed by patients, tolerating uncertainty, regulating fear, negotiating family dynamics, and repeatedly confronting mortality, remains comparatively invisible (Hochschild 1983; Mishel 1988; Frank 1995; Lupton 2012; Breitbart 2017). In this case, early engagement required sustained emotional regulation and narrative work that accrued cumulative internal cost over time.

Capacity debt introduces a temporal lens that helps distinguish capacity decline from capacity allocation. Although disease progression inevitably reduces baseline reserves (Murray et al. 2005; Berger et al. 2015), capacity debt explains why disengagement may occur earlier than illness severity alone would predict. Two patients with similar disease trajectories may exhibit different engagement patterns depending on how intensively capacity was expended earlier in illness, as illustrated conceptually in Figure 1.

Importantly, disengagement in this case was selective rather than global. The patient withdrew from emotionally demanding discussions but continued to participate coherently in concrete decisions. This selectivity supports the interpretation of disengagement as conservation rather than avoidance. Misinterpreting such withdrawal as psychopathology risks unnecessary escalation of psychosocial interventions and may further deplete remaining capacity.

The case also highlights relational and systemic consequences of unrecognized capacity debt. Family members may interpret withdrawal as emotional disengagement or loss of hope, increasing pressure for continued conversation when capacity is most depleted. Clinicians, in turn, may experience moral distress when patients no longer engage in ways that previously signaled “good care” (Rushton 2017). Recognizing capacity debt may, therefore, mitigate relational strain and clinician distress by reframing disengagement as ethically intelligible.

This case also raises questions about how contemporary health systems may inadvertently generate capacity debt. Early serious illness communication and repeated goals-of-care discussions are increasingly embedded within quality metrics and institutional pathways. While intended to promote patient-centered care, such frameworks often privilege frequency and early timing without accounting for cumulative cognitive and emotional cost. In this context, disengagement may represent not individual vulnerability, but a predictable consequence of system-level expectations that treat engagement as endlessly renewable. Capacity debt, therefore, invites reflection on how quality in palliative care is defined and measured.

From an ethical perspective, capacity debt complicates traditional understandings of autonomy. Respect for autonomy is often operationalized as eliciting preferences and promoting participation (Elwyn et al. 2012). This case suggests autonomy has a longitudinal dimension: excessive early demands may erode future decisional freedom. Ethical palliative care thus requires not only honoring expressed preferences, but also stewarding patient capacity over time to preserve meaningful agency (Beauchamp and Childress 2019).

A further dimension of capacity debt concerns patients’ anticipatory relationship with their future selves. Some patients engage intensively early in illness to settle decisions, protect family members, or preserve dignity while they still feel able. In doing so, they may knowingly or unknowingly draw upon reserves they sense will not be available later. Capacity debt thus reflects not only cumulative burden, but also a form of temporal self-sacrifice, deepening the ethical relevance of pacing.

Clinically, this case underscores pacing as a core palliative care skill. Pacing involves attending to what conversations occur, when they occur, and at what cumulative cost. Allowing silence, tolerating unfinished conversations, and deferring revisiting topics may protect remaining capacity without compromising care quality.

## Conclusion

This case demonstrates how late disengagement in palliative care may arise from cumulative depletion of patient capacity following early intensive engagement rather than from psychopathology or loss of insight. Interpreting this pattern as capacity debt offers a non-pathologizing and ethically grounded framework that preserves respect for patient autonomy while acknowledging its longitudinal vulnerability. Pacing thus emerges as a core clinical and ethical skill in palliative care, enabling engagement to remain supportive rather than depleting across the illness trajectory.

Recognizing capacity debt reframes late disengagement not as failure or pathology, but as an ethically intelligible consequence of cumulative care demands, positioning pacing as a core clinical skill in palliative and supportive care.

## Data Availability

All data generated or analyzed during this study are included in this published article.
